# Mimicking orchids lure bees from afar with exaggerated ultraviolet signals

**DOI:** 10.1002/ece3.9759

**Published:** 2023-01-29

**Authors:** Daniela Scaccabarozzi, Klaus Lunau, Lorenzo Guzzetti, Salvatore Cozzolino, Adrian G. Dyer, Nicola Tommasi, Paolo Biella, Andrea Galimberti, Massimo Labra, Ilaria Bruni, Giorgio Pattarini, Mark Brundrett, Monica Gagliano

**Affiliations:** ^1^ School of Pharmaceutical Science and Technology Tianjin University Tianjin China; ^2^ School of Molecular and Life Sciences Curtin University Bentley Western Australia Australia; ^3^ Institute of Sensory Ecology Heinrich‐Heine University Düsseldorf Düsseldorf Germany; ^4^ ZooPlantLab, Dipartimento di Biotecnologie e Bioscienze University of Milano – Bicocca Milan Italy; ^5^ Department of Biology University of Naples Federico II Naples Italy; ^6^ Bio‐Inspired Digital Sensing Lab, School of Media and Communication RMIT University Melbourne Victoria Australia; ^7^ Department of Physiology and Neuroscience Program, Biomedicine Discovery Institute Monash University Clayton Victoria Australia; ^8^ Department of Mathematics and Physics University of Stavanger Stavanger Norway; ^9^ School of Biological Sciences University of Western Australia Perth Western Australia Australia; ^10^ Biological Intelligence (BI) Lab, Faculty of Sciences & Engineering Southern Cross University Lismore New South Wales Australia; ^11^ Sydney Environment Institute (SEI) The University of Sydney Camperdown New South Wales Australia

**Keywords:** bee sensory ecology, ecological interactions, flower attraction, food deception, orchid floral mimicry, pollination success, salient stimuli, ultraviolet reflectance, visual food deception

## Abstract

Flowers have many traits to appeal to pollinators, including ultraviolet (UV) absorbing markings, which are well‐known for attracting bees at close proximity (e.g., <1 m). While striking UV signals have been thought to attract pollinators also from far away, if these signals impact the plant pollinia removal over distance remains unknown. Here, we report the case of the Australian orchid *Diuris brumalis*, a nonrewarding species, pollinated by bees via mimicry of the rewarding pea plant *Daviesia decurrens*. When distant from the pea plant, *Diuris* was hypothesized to enhance pollinator attraction by exaggeratedly mimicking the floral ultraviolet (UV) reflecting patterns of its model. By experimentally modulating floral UV reflectance with a UV screening solution, we quantified the orchid pollinia removal at a variable distance from the model pea plants. We demonstrate that the deceptive orchid *Diuris* attracts bee pollinators by emphasizing the visual stimuli, which mimic the floral UV signaling of the rewarding model *Daviesia*. Moreover, the exaggerated UV reflectance of *Diuris* flowers impacted pollinators' visitation at an optimal distance from *Da. decurrens*, and the effect decreased when orchids were too close or too far away from the model. Our findings support the hypothesis that salient UV flower signaling plays a functional role in visual floral mimicry, likely exploiting perceptual gaps in bee neural coding, and mediates the plant pollinia removal at much greater spatial scales than previously expected. The ruse works most effectively at an optimal distance of several meters revealing the importance of salient visual stimuli when mimicry is imperfect.

## Graphical abstract

Donkey orchids mimic and exaggerate UV reflectance signals of rewarding model pea plants to attract pollinators. The ruse works exclusively from afar when the orchid is at an optimal distance away from the pea it mimics. Our findings explain the adaptive significance of salient UV display in long‐range plant‐bee communication and why deceptive plants succeed despite imperfect floral mimicry.

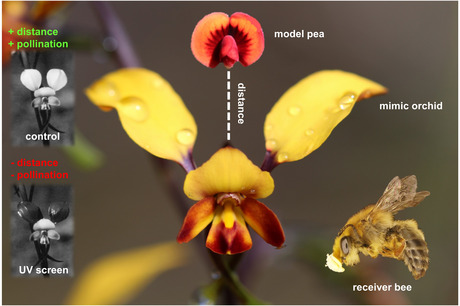

## 1 INTRODUCTION

The art of deception, involving a range of strategies individuals adopt to change the perception and behavior of others, is commonly practiced by many organisms across the animal and plant kingdoms. Mimicry, a form of deception, allows individuals to conceal their identity and avoid recognition by (more or less) closely imitating the behavior or resembling the appearance of their models (Dawkins & Krebs, 1979). One of the most remarkable examples of these deceptive adaptations is the duping of pollinating animals by plant mimics. Among the 32 families of deceptive plants (Renner, 2006), orchids are undoubtedly the master tricksters. With an estimate of about one‐third of all species lacking floral reward to pollinators (Ackerman, 1986a; Dafni, 1984; Jersáková et al., 2006), orchids deceive by luring food‐seeking animals by fine‐tuned mimicry (i.e., Batesian floral mimicry) or general resemblance of rewarding flowers (i.e., generalized food deception; Shrestha et al., 2020). Surprisingly, how plants succeed in their deception despite widespread imperfect mimicry remains poorly understood (Roy & Widmer, 1999; Schiestl, 2005; Vereecken & Schiestl, 2008). In animals, the success of imperfect mimicry has been explained by high‐salience traits, which overshadow other “less important” traits (Cuthill, 2014; Kazemi et al., 2014) by being highly discriminable from the background (Frieman & Reilly, 2015). Although high‐salience of signals such as attention‐grabbing colors and visual patterns occur as frequently in animals (Kazemi et al., 2014) as in plants (Jersáková et al., 2012; Peter & Johnson, 2008, 2013), their role in explaining imperfect mimicry in plants has received comparatively less attention (Vereecken & Schiestl, 2008). In this study, we examined the role salient ultraviolet (UV) signaling plays in the imperfect floral mimicry of a rewardless orchid that falsely advertises a reward to attract bees when afar from model plants.

Flowering plants and pollinating insects interact through a wide range of sensory modalities, which affect both the pollinator's foraging behavior and the plant's reproductive success (Glover, 2011; Leonard et al., 2011a). Pollinating insects, in particular bees, make their foraging decisions most effectively by combining visual, olfactory, and somatosensory floral signals (Kulahci et al., 2008; Leonard et al., 2011a), yet their innate preference for conspicuous floral displays usually makes color and contrasting visual patterns the primary means by which plants first attract them (Naug & Arathi, 2007; van der Kooi et al., 2019). Bees, the main flower visitors, have phylogenetically conserved trichromatic vision (Briscoe & Chittka, 2001), which can be conveniently modeled with maximum sensitivity UV (approx. 340 nm), Blue (435 nm) and Green (560 nm) photoreceptors (Chittka & Kevan, 2005). Plants produce striking floral markings and patterns by absorbing and reflecting UV light (Briscoe & Chittka, 2001; Dinkel & Lunau, 2001; Lunau et al., 2006, 2021; Papiorek et al., 2016). Interestingly, it is the UV reflectance display rather than the UV pattern (absorbance and reflectance) that increases insect visitation (Johnson & Andersson, 2002; Klomberg et al., 2019). The high chromatic contrast that such UV signals can generate is thought to enhance color salience in an opponent color system (Chittka et al., 2001; Lunau et al., 2006; Papiorek et al., 2016); however, such chromatic contrast is assumed to work only at relatively short distances of about few centimeters (e.g., UV absorbing “floral guides”; Garcia et al., 2021; Giurfa et al., 1996; Horth et al., 2014; Orbán & Plowright, 2014). This is because bees typically only use the long wavelength green input channel of their visual system to enable fast achromatic processing and detection of small target signals (Klomberg et al., 2019), although some psychophysics shows that alternative chromatic channels may in some cases also be important for bee detection and recognition (Dyer et al., 2019; Morawetz et al., 2013; Zhang et al., 1995). That UV reflectance can also attract pollinator insects from further afield has been posited for decades (Burr et al., 1995; Daumer, 1956, 1958; Koski & Ashman, 2014) but remains unverified.

Salient UV signals against the background may be particularly relevant for increasing long‐distance attractiveness in plants that employ flower mimicry (Dyer, 1996), but the question of their effectiveness is not easily testable because of the flower structures that incorporate many color tones together. To obtain experimental access to this question, it is possible to focus on modulating signals in flowers that display salient UV signals. One such plant is the Australian donkey orchid *Diuris brumalis* whose two outer petals appear yellow to human vision and also strongly reflect UV that would be conspicuous to the visual system of bees (Burr et al., 1995). *Diuris brumalis* is a food‐deceptive species, which secures pollination by resembling the co‐occurring rewarding pea plant *Daviesia decurrens* (Scaccabarozzi et al., 2018). The mimicry signals consist of both color reflectance and inner flower shape, as the outer petals diverge from the pea flower shape (Scaccabarozzi et al., 2018). Whilst the mimicry in size and shape is imperfect, the orchid coloration, with the average color loci corresponding to the UV region, is perceptually similar to the pea model in color space; such overlap (<0.06 color hexagon units) makes the two species not readily distinguishable in the eyes of their bee pollinator, *Trichocolletes* spp. (Hymenoptera: Collectidae; Scaccabarozzi et al., 2018). Food‐deceptive orchids are known for gaining their pollination success not only by resembling a specific rewarding model flower (Dyer et al., 2012; Scaccabarozzi et al., 2018; Schaefer & Ruxton, 2009), but also exaggerating their floral signals that advertise the false reward and thus increase pollinator responses (Ackerman, 1986b). Therefore, we hypothesized that the two outer petals of *Diuris* function as an exaggerated version (for UV reflectance display) of the floral signal display that *Trichocolletes* bees normally encounter in the rewarding *Daviesia* peas. We expected that modulating the exaggerated UV signals of *Diuris* over a spatial scale would affect pollinia removal when orchids are relatively distant from their model food plants because pollinators are more likely to mistake the orchid for the rewarding model when afar. In order to setup the UV modulation experiments on the distance range that is ecologically relevant for the orchid mimicry success, our study firstly describes the function of pollinia removal in orchids according to their distance from the model pea plants.

## 2 MATERIALS AND METHODS

### 2.1 Study system

Endemic to Western Australia, the orchid *Di. brumalis* produces yellow–brown nectarless flowers between July and August and is pollinated via mimicry of rewarding pea plants (*Daviesia* spp.) by native *Trichocolletes* (Colletidae) bees (Scaccabarozzi et al., 2018; Scaccabarozzi, Guzzetti, et al., 2020; Scaccabarozzi, Dixon, et al., 2020; Houston et. al., in press). *Trichocolletes* is a genus of solitary bees that are specialist and speed (visits last <2 s) feeder on pea flowers and display a distinctive and identical behavior on both orchids and peas, confirming that it is successfully deceived. The orchid mimics the papilionaceous flower typical of the pea model and while the visible spectrum differs between the mimic and model flower, they are likely to look similar through a bee visual model (Scaccabarozzi et al., 2018). However, the orchid flower diverges from the pea flower structure by exhibiting two prominent outer petals.

We carried out our study in *Di. brumalis* populations spread along the Darling Range in Western Australia during 2018, 2019, and 2020 (Table S1). In situ studies and experimental settings were preferred as the orchids are protected by national regulation and their withdrawal is only allowed for few biological materials.

### 2.2 Floral morphology and color properties

To test the hypothesis that the two outer petals of *Diuris* may function as an exaggerated version of *Daviesia* floral signals, we firstly determined whether the outer petals were the component of the *Diuris* flower with the highest UV spectral reflectance so amplifying the UV reflectance of the pea model. We obtained UV measurements for each floral component (*n* = 6 flowers) for both orchid and pea plants using a Cary 4000 UV–Vis spectrophotometer (Agilent Technologies) and calculating the average spectral reflectance for each floral part.

Secondly, we measured the size of the flower components of the flower (mid‐inflorescence flower) in 10 plants of both *Diuris* and *Daviesia* (Figure S1, Data S1). We obtained for both species a UV‐salient signal according to the cut value of Australian flowers following Dyer (1996) (Data S1). Flower components' area was estimated as follows: as flowers of *Diuris* and *Daviesia* show little concavity or convexity, the areas of the outer and central floral components of *Diuris* were estimated by approximating the components to the closest geometric figures, the ellipse (orange) and the circle (green), respectively (Figure S1). *Daviesia* standard petals' area was approximated to an ellipse, to which was subtracted a secondary minor ellipse circumscribing the wing and keel petals (Figure S1, Data S1).

To quantify the contrast of the respective flower signals, we used the bee visual parameters according to Chittka and Kevan (2005) and neural coding that enables converting visual signals sensed by each receptor channel into Excitation values between 0 and 1.0. The visual system was adapted to foliage background with a biologically relevant neural resting excitation value of 0.5 and a contrast of zero (Chittka et al., 1994; Spaethe et al., 2001). This model enables the calculation of absolute contrast values ranging from 0 to 0.5 (maximum contrast) for any stimulus that is different from the background as perceived by the visual system of bees (Table 1).

**TABLE 1:** Average of excitation values (±SD, standard deviation) of bee photoreceptors (UV, blue, green) according to Chittka (1992) and Chittka et al. (1994) and relative corrected values for *Diuris* and *Daviesia* flower components as shown in Figure 1, including *Diuris* outer petals treated by UV filter.

Flower components		*E* (uv) ± SD	*E* (uv)‐0.5	*E*(b) ± SD	*E*(b)‐0.5	*E*(g) ± SD	*E*(g)‐0.5
1	*Diuris brumalis* outer petal	0.84 ± 0.03	0.34	0.49 ± 0.07	0.01	0.70 ± 0.03	0.20
	*Di. brumalis* outer petal treated with UV filter	0.48 ± 0.03	0.02	0.32 ± 0.07	0.18	0.70 ± 0.03	0.20
2	*Di. brumalis* dorsal sepal	0.77 ± 0.09	0.27	0.40 ± 0.09	0.10	0.57 ± 0.07	0.07
3	*Di. brumalis* lateral labellum lobe	0.64 ± 0.17	0.14	0.20 ± 0.11	0.30	0.42 ± 0.17	0.08
4	*Di. brumalis* labellum	0.25 ± 0.17	0.25	0.07 ± 0.07	0.43	0.15 ± 0.03	0.35
5	*Daviesia decurrens* standard petal	0.77 ± 0.02	0.27	0.39 ± 0.09	0.11	0.45 ± 0.06	0.05
6	*Da. decurrens* wing petal	0.56 ± 0.10	0.06	0.13 ± 0.05	0.37	0.14 ± 0.06	0.36

*Note*: Excitation values range between 0 and 1.0 where a value of 0.5 represents no excitation of the sensory neural channel, and so, the absolute maximum excitation contrast is 0.5 for each respective channel.

False color photography in “bee view” format was used to reveal the overall color pattern perceived by bees of *Diuris* and *Daviesia* flowers (Figure 2a,b; Methods S1,S2). Spectrometer measurements of flower components of *Diuris* and *Daviesia* were converted according to the established bee visual model (Chittka, 1992). The location of color loci was calculated from the mean of reflectance for floral parts of *Di. brumalis* and *Da. decurrens* (Figure 2c).

### 2.3 Model‐mimic distance experiment

To test whether *Diuris* pollination success varies depending on the distance to the model pea plants, in 2019, we first quantified the distance between an individual orchid and all the surrounding pea models within a quadrat of 30 × 30 m centred on a single orchid plant (*N* = 122 orchids across five populations; Table S1, Figure S2) for all orchid plants per population. As a result, all quadrats overlapped within the same population but not among populations (as the distance between populations was >500 m). To quantify pollination attraction, we recorded the number of pollinia removed by pollinators in all orchids per population (pollinia removed in orchids were counted by visually observing the lack of pollinia at the top of the column), recording the number of flowers per plant in both orchids and pea plants. We analyzed the distance data by using a Generalized Mixed Effect Model (GLMM) with the Poisson distribution. The response variable in the model was the number of pollinia removed and the fixed effects were the distance from the nearest pea model and the number of orchid flowers. The population was treated as a random factor since it was found to be significant in influencing the number of pollinia removed. The model was evaluated for its dispersion parameter and residuals were evaluated for the assumption of overdispersion and homoscedasticity.

### 2.4 Ultraviolet manipulations experiments

Subsequent manipulation experiments were carried out in the field in 2019 and 2020 by screening the UV properties of the two *Diuris* outer petals with a UV filter solution (Johnson & Andersson, 2002; Peter & Johnson, 2013), which effectively eliminates UV reflectance whilst transmitting all wavelengths above 400 nm (Figure 2a,b). To confirm that treated *Diuris* outer petals did not excite the UV bee photoreceptor as untreated orchid petals and *Daviesia* petals did, we analyzed the spectral reflectance measurements for the different floral components using the model of bee vision including treated petals (Chittka, 1992; Table 1). False color photography in “bee view” format was applied on *Diuris* flower with treated outer petals to show the overall color pattern (Figure 2b).

The effect of the UV reflectance filter solution (Kinesys) on the number of *Trichocolletes* bee visits to *Diuris* orchids was tested using choice experiments (Methods S1; Data S3) to rule out the potential effect of the UV filter solution on attracting or repelling bee pollinators.

In the first field manipulation experiment in 2019, we tested the hypothesis that UV reflectance enhances orchid pollination success (pollen removal) only when orchids are out of the patch of model pea plants as we expected that when orchids are relatively distant from their model food plant pollinators are more likely to mistake the orchid for the rewarding model per conditioning effect. Accordingly, we quantified the number of pollinia removed from *Diuris* flowers by free‐foraging bees when the mimicking orchid occurred inside [IN] and outside [OUT] the 30 × 30 m patch of model plants (within a maximum distance of 10 m from the patch; Figure 3a). The patch size encompassed most orchid plants belonging to an individual population according to former studies on male reproductive success (proxy) of *Diuris* at this location (Scaccabarozzi et al., 2018). Over a 4‐day period, all orchids in both [IN] and [OUT] groups (*N* = 400 across five populations, Table S1) were treated with the UV filter. Within each group, a randomly selected half of the orchids was sprayed on the front and back of the two outer petals (treatment, T) and the other half of the orchids at the base of the corolla (control, C). Number of flowers was standardized in each clump by removing flowers in excess to obtain the same number of flowers in treated and control flowers to allow comparison of the flower display. The UV filter was applied before the daily peak of bee activity and from 11.00 a.m. to 1.00 p.m. and during the subsequent 2‐h period (corresponding to the filter persistence on petals) from 1.00 to 3.00 p.m. we recorded the number of pollinia removed from the orchids within each group. Prior to the UV filter application, the treated and untreated plants were numbered and tagged. We also recorded the number of pollinia already removed per flower/per plant to make sure of the net counting of pollinia. When revisiting the plants for scoring pollinia, we checked the plants in the same order followed prior to the treatment. Statistics were based on comparisons of removed pollinia between experimental groups (UV‐treated petals) and control groups (UV‐untreated petals).

In the second field manipulation experiment, in 2020, we tested the hypothesis that by displaying an exaggerated version of *Daviesia*'s attractive UV reflectance, *Diuris* benefits from pollinators that mistake it for the rewarding model from afar. We quantified pollinia removal within 63 orchid groups randomly selected across three large orchid populations (Populations 1, 2, 3; Table S1). Each orchid group consisted of two orchid clumps, each containing between 2 and 12 plants. Each orchid clump was selected to be at approximately the same distance from a model pea plant (from 0 to 15 m) at a variable angle from the pea plant (Figure 4a).

Within each orchid clump, *Diuris* floral display (i.e., number of flowers in each clump) was balanced by removing flowers in excess to make the sample size the same. This was made randomly to compare always the same floral display between treated and untreated orchid clumps and to control potential bias due to the attraction to an unbalanced floral display. Within each group, the UV filter solution was sprayed on the outer petals of one clump (treatment, T) and at the base of the corolla on the other clump (control, C) as in the previous experiment (same treatment and plant visitation timing). Prior to the UV filter application, the treated and untreated plants were numbered, tagged, and the number of pollinia removed per flower/per plant was recorded. Pea plant flower range was uniform among plants at the time of the experiment (according to categories in Scaccabarozzi et al., 2018; see Data S6). The number of pollinia removed from the UV‐treated and control orchids within each group was recorded as a function of the orchid's distance to the nearest pea plant and was modeled by a Poisson GLMM (appropriate for count data) with a fixed effect for treatment. The number of orchid flowers was included as a covariate in the model.

## 3 RESULTS

### 3.1 Contrasting floral displays of models and mimics

**FIGURE 1:**
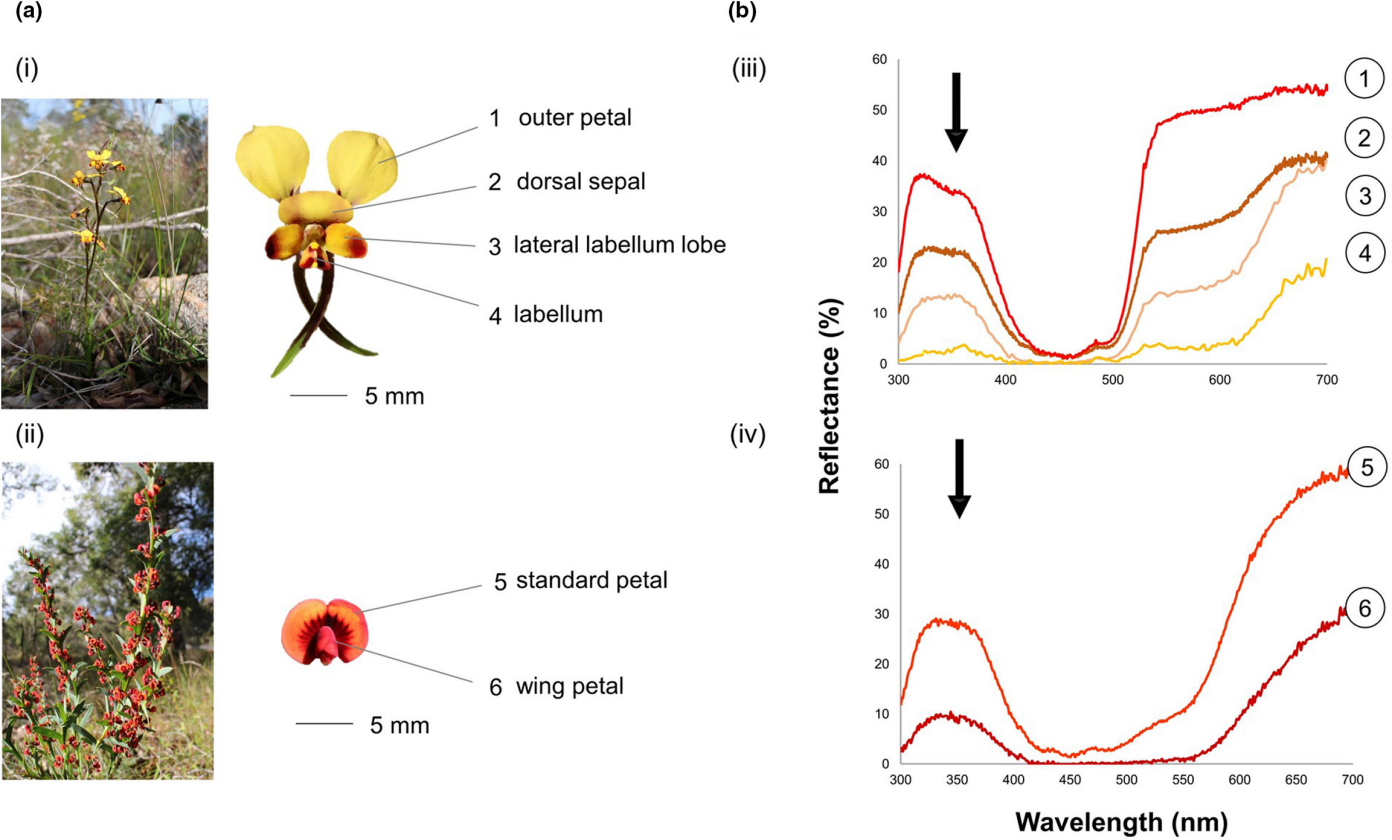
Floral morphology and color properties of the mimicking orchid and its pea model. (a) Flower morphology of the orchid *Diuris brumalis* (i) and the pea, *Daviesia decurrens* (ii). The dorsal sepal, labellum lateral lobes, and the labellum in *Diuris* flower and standard petal and wing petal of *Daviesia*. The outer petals in the orchid are the component of the floral architecture that is absent in the pea. (b) Average color reflectance measured on flower components in *Diuris* (iii) and *Daviesia* (iv) peaks in the UV bands (black arrows). Color reflectance in the UV wavelengths (300–400 nm) varied between 0.5% and 37% in *Diuris* sepals and petals and 2.5% and 28% in the pea model. The UV reflectance of *Diuris* outer petals ranged between 18% and 37% (see Data S1 and S2).

**FIGURE 2:**
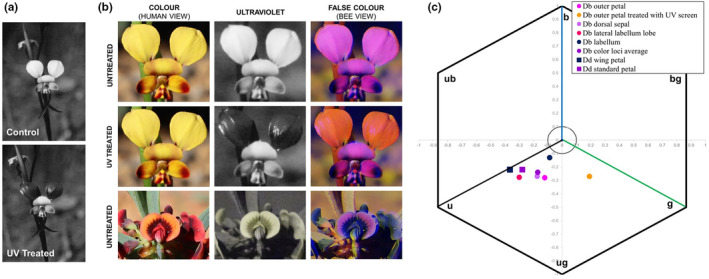
Color patterns perceived by bees in treated and untreated *Diuris* flowers and untreated *Daviesia*. (a) *Diuris* flower photographed in UV before (control, C) and after applying the UV filter on the outer petals (UV treated, T). (b) False color photography in “bee view” reveals the overall color pattern perceived by bees in treated (i.e., application of the UV filter solution) and untreated outer petals of *Diuris* flower and untreated *Daviesia* flower. The UV filter is effectively a long‐pass filter transmitting all wavelengths above 400 nm, free of fragrance, oil, PABA, alcohol, parabens, and preservatives (Kinesys). Importantly, the UV images of treated outer petals show very similar reflectance properties to the background and stem foliage reflectance, confirming that the experimental manipulation knocked out UV signaling with respect to background coloration. (c) Location of color loci was calculated from the mean of reflectance for floral parts of *Diuris brumalis* (Db), and *Daviesia decurrens* (Dd). The calculations were made using the Hexagon color model of bee vision (Chittka, 1992). This model represents the internal perception of flower colors by bee pollinators, and resultant sectors (u [ultraviolet]; ub [ultraviolet‐blue]; b [blue] bg [blue‐green]; g [green]; ug [ultraviolet‐green]) show how bees likely interpret spectral signals].

The size of the orchid flower is about three times bigger than the pea flower (Figure 1a). The outer petals proved to be both the largest component of the orchid flower and the area with the highest UV reflectance (Figure 1b; Figure S1, Data S1). The strength of the UV signaling in *Diuris* had a contrast value of 0.34, which is 26% greater than the UV channel contrast value of 0.27 in *Daviesia* standard petals (Table 1). False color photography in “bee view” revealed the similarity of the overall color pattern perceived by bees of *Diuris* and *Daviesia* flowers (Figure 2b).

According to the color model, the petals of *Diuris* and petals of *Daviesia* are located in the bee‐perceived “ug” (UV‐green) and “u” (ultraviolet) sectors of the Hexagon color space related to the excitation of bee photoreceptors and subsequent bee neural coding of information (Figure 2c, Table 1; see Chittka, 1992; Chittka et al., 1994).

### 3.2 Orchid pollinia removal relates to mimic‐model distance

Mimic‐model distance on large scale revealed that the number of pollinia removed from the orchid flowers decreased significantly with the distance between orchid and pea (Figure S2, Data S4). Specifically, pollinia removal decreased significantly with orchids' distance from the pea model (*χ* ^2^ = 10.34, *p* = .001) while it was found a positive logarithmic dependency with the number of orchids' flowers (*χ* ^2^ = 10.75, *p* = .001).

### 3.3 UV manipulations experiments and orchid success in model plants over distance

The UV filter treatment had no attracting or repelling effect on the pollinators (see Methods S1, Data S3) confirming the pollinator visits were independent from the mean used to screen the UV signal (UV screening spray). Treated petals of *Di. brumalis* are located in the bee‐perceived “g” (green) Hexagon sector and according to Scaccabarozzi et al. (2018) did not excite bee UV photoreceptors (Figure 2c, Table 1). Secondly, the color model corroborated that the excitation of Green receptor, which is known to be important for how bees efficiently find flowers (Giurfa et al., 1996; Skorupski & Chittka, 2010; Garcia et al., 2021), was not affected by UV filter treatment (Table 1). False color photography in “bee view” confirmed that the UV filter knocked out UV signaling with respect to background coloration (Figure 2b).

**FIGURE 3:**
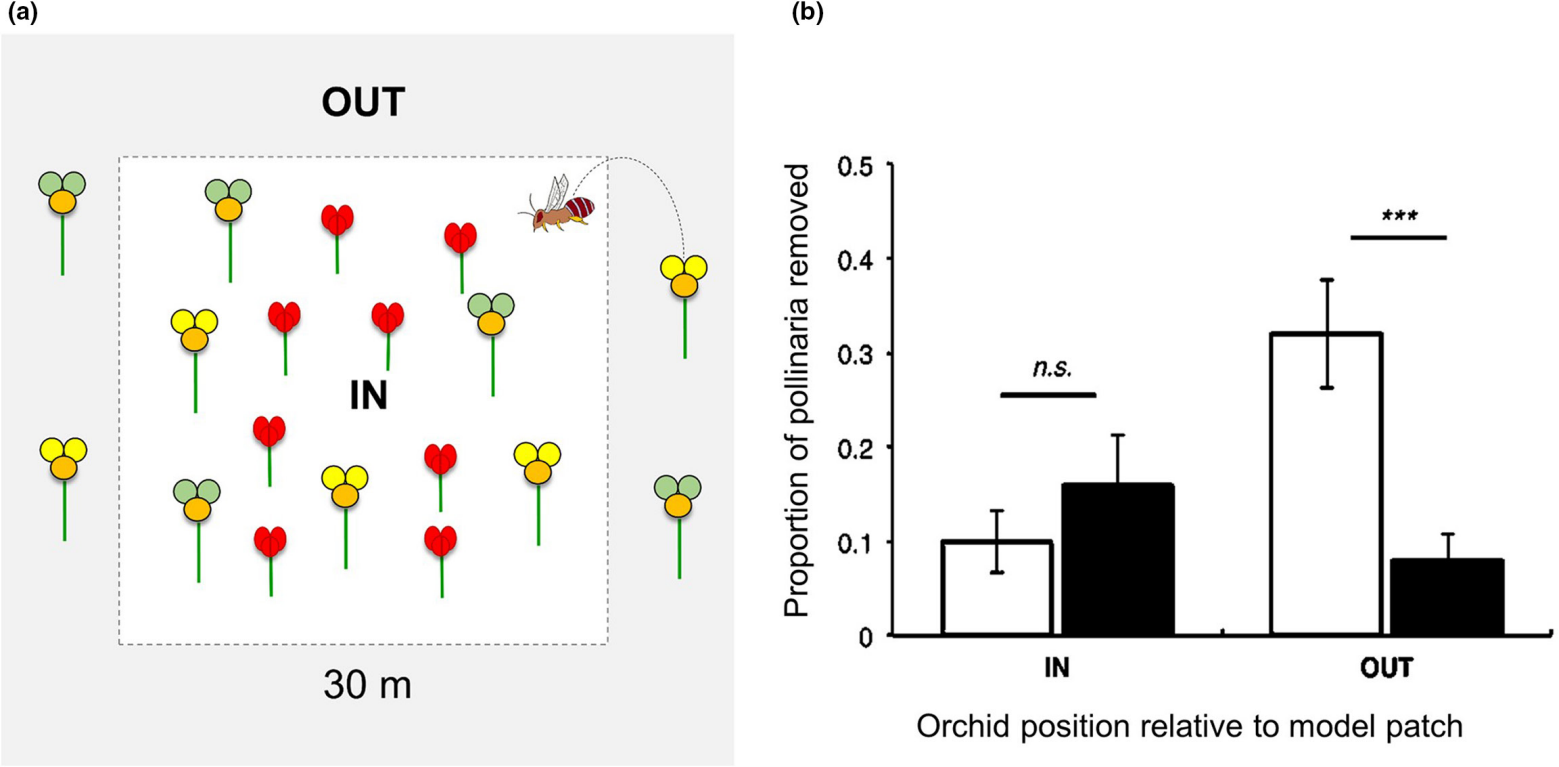
Effect of distance from model plants on *Diuris* pollinia removal. (a) *Diuris* orchids (yellow [untreated] and green [UV‐treated] flowers) inside [IN] and outside [OUT] a 30 × 30 m patch with *Daviesia* pea (red flowers). (b) Mean proportion of pollinia bees removed from treated (black bars) and untreated *Diuris* flowers (white bars) relative to the orchid's distance ([IN] and [OUT]) from the model pea. Each experimental group consists of *N* = 100 orchids. Error bars are 95% confidence intervals; n.s., no significant difference among experimental groups; ***Significant difference at Bonferroni‐corrected *α* = .0125.

In the first field manipulation experiment, we quantified the number of pollinia removed from treated and control *Diuris* flowers by free‐foraging bees when the mimicking orchid co‐occurred with the model pea within a 30 × 30‐m patch per orchid population [IN] and when the mimics occurred outside the patch of model plants [OUT] (Figure 3a; Data S5). The application of the UV filter on the two outer petals resulted in a significant effect on the number of pollinia removed by bees from the orchid flowers (*χ* ^2^ = 19.81, *p* < .001). There was no difference in the pollinia removal of *Diuris* whose outer petals had been treated with the UV filter [IN‐T] compared with untreated control orchids [IN‐C] inside the patches of model plants (Figure 3b). Outside the patches of model plants, however, orchids with UV filter treatment [OUT‐T] experienced significantly lower pollinia removal than control ones [OUT‐C] (Figure 3b).

**FIGURE 4:**
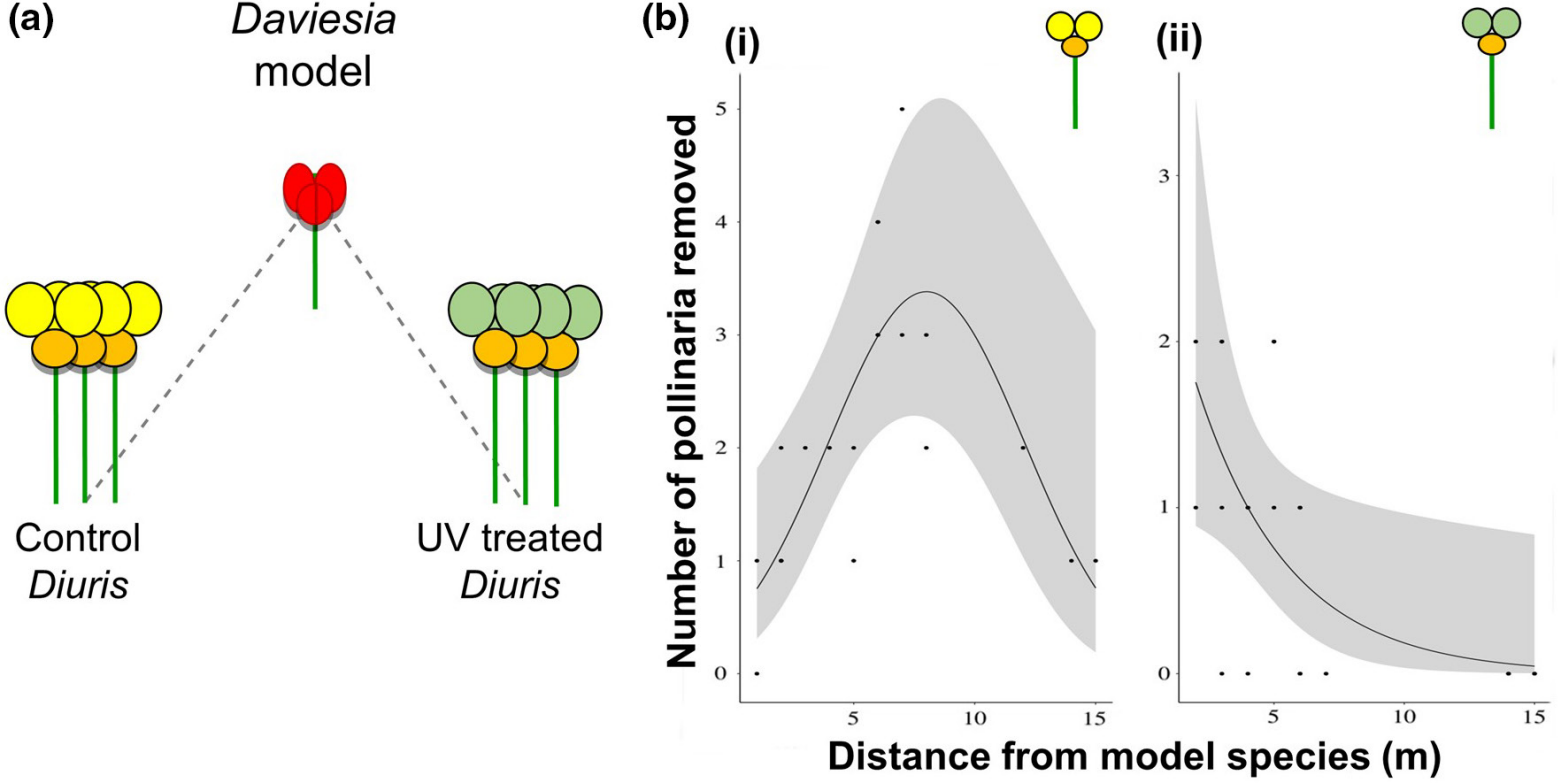
Effect of *Diuris* UV reflectance on the orchid's pollinia removal relative to mimic‐model distance. (a) Experimental setup treated, control orchid groups and pea plant, (b) Pollinia removal was quantified in 195 orchids (*N* = 476 orchid flowers). Pollinia removal of control *Diuris* relative to distance from *Daviesia* (i) was best described by an inverted parabolic function peaking at ~8 m distance from model pea (*χ* ^2^ = 9.87, *p* < .05 for the squared and linear term, respectively) (*N* = 238 flowers, *n* = 43 pollinia removed). Pollinia removal of UV‐treated orchids (ii) exhibited an exponential decrease with distance from model pea plants (*χ* ^2^ = 10.26, *p* < .001) (*N* = 238 orchid flowers, *n* = 17 pollinia removed). Refer to Data S6 for full data.

In the second field manipulation experiment, we found that pollinia removal of control *Diuris* increased with distance by peaking at ~8 m away from the model peas before declining and becoming ineffectual at distances >15 m (Figure 4b(i); Data S6). The effect of the number of flowers was found to be not significant (*χ* ^2^ = 0.73, *p* = .74) and the covariate was subsequently removed. We detected no effect of UV reflectance on *Diuris* pollinia removal when the orchids were closer than a few meters to their model pea plants (Figure 4b(i, ii); Data S6).

## 4 DISCUSSION

Our results establish that *Diuris* orchids mimic and exaggerate *Daviesia*'s attractive floral signals in terms of UV reflectance, display, and contrast as generally perceived by bee pollinators. Spectral reflectance and morphological measurements of flower components confirmed that *Diuris* functioned as an exaggerated version of the floral signals bees normally encounters in the rewarding *Daviesia* peas. Flowers that reflect >10% UV radiation, like *Diuris* and *Daviesia*, are shown to have evolved this salient trait to likely improve communication with bees since most organic background material like leaf foliage has very low UV reflectance (Chittka et al., 1994; Dyer, 1996; Spaethe et al., 2001; van der Kooi et al., 2019).

Field manipulation experiments showed that the exaggerated UV signal of *Diuris* outer petals enhances the orchid pollinia removal. By masking the UV reflectance in half of the orchids inside the *Daviesia*'s patch, there was no difference in the pollinia removal of *Diuris* whose outer petals had been UV screened [IN‐T] compared with untreated control orchids [IN‐C] inside the *Daviesia*'s patch (Figure 3b). At closer range, within pea patch, bees apparently recognize plants by spotting other visual traits as the shape of *Diuris* two outer petals. A color trait may become less effective in ensuring successful mimicry when other secondary traits such as size and shape of the flowers can be better discriminated (Gigord et al., 2002; Johnson & Morita, 2006). Outside the model patch, however, orchids with UV filter treatment [OUT‐T] experienced substantially lower pollinia removal than control ones [OUT‐C] (Figure 3b), due to a lack of the salient signal, which is associated with the model trait. Thus, the exaggerated UV signal produced by *Diuris* outer petals only increased the orchid's pollinia removal when the mimic was further away from its models' patch. Our findings demonstrate that salient floral UV reflectance plays a critical role in ensuring *Diuris* pollinia removal and explain why the exaggerated UV signal is strategically relevant in floral mimicry when the model is not very close to the mimic. According to previous theories predicting the effectiveness of the mimic's floral stimuli to decline with distance from its model (Duffy & Johnson, 2017; Johnson & Schiestl, 2016), we also found that the number of pollinia removed from the orchid flowers decreased significantly with the distance between orchid and pea (Figure S2). However, the strength and direction of this effect may vary across different spatial scales, and conclusions about the importance of floral stimuli will depend on the scales at which studies are undertaken. For example, by examining the mimic‐model effect at considerably smaller spatial scales than usually investigated (i.e., tens to hundreds of meters) (Duffy & Johnson, 2017; Johnson et al., 2003; Peter & Johnson, 2008), our results suggest that the exaggerated UV reflectance of *Diuris* outer petals function to enhance pollination at an optimal model‐mimic range of ~8 m. *Diuri*s outer petals might promote pollinator deception via bee cognitive misclassification (Dyer et al., 2012; Johnson & Schiestl, 2016), displaying color frequencies below the optimal range of color discrimination in hymenopteran (i.e., 400–500 nm) (Peitsch et al., 1992), especially for free‐flying honeybees (Rohde et al., 2013; von Helversen, 1972). However, these findings might be context dependent and be specifically linked to the spatial distribution and abundance of the model species for *Diuris*; we expect that the optimal model‐mimic range may vary when involved model species characterized by different distribution and density.

But why might the observed distance range from model species be optimal? To understand this question, we must delve into both the neurophysiology and physiology of how bee pollinators perceive their world. When a bee receives sweet tasting nectar reward from a rewarding plant like *Da. decurrens*, this promotes a sustained positive neural response via the ventral unpaired median (VUM) neurons that permit an association between flower and reward with a sustained spiking response of about 15 s (Hammer, 1993; Perry & Barron, 2013), and can enable simple associative learning of color information (Dyer & Chittka, 2004; Giurfa, 2004). It is also known that precise color memory in both bees and humans requires simultaneous viewing conditions that decay in less than a second once a target model is no longer in view (Dyer & Neumeyer, 2005; Uchikawa & Ikeda, 1981); therefore, being close to a model species might allow a bee to identify potential differences that unmask the deception (von Helversen, 1972). Given that bees may fly up to about 7 m in a second (Spaethe et al., 2001; Srinivasan & Lehrer, 1985), we hypothesize the 8 m distance we observed for optimal pollinia removal is beyond the theoretical upper limit where precise color vision operates; at such distances, the bee has to recall from memory what it thought was rewarding and tends to prefer a slightly more salient comparative stimulus, an effect related to peak shift discrimination (Leonard et al., 2011b; Lynn et al., 2005; Martínez‐Harms et al., 2014). The fast visits of *Trichocolletes* bees on both model and mimic flowers (Scaccabarozzi et al., 2018), suggest that *Diuris* benefits from foraging speed behavior that unfavours the accuracy of bee choices (Chittka et al., 2003). Thus, we propose that orchids like *Diuris* master deception by employing both exaggerated signaling and by exploiting the perceptual gaps in pollinators' visual processing.

Our results also highlight that we gain a very different understanding of the relative role of floral signals if we work at one scale over another and consider the dynamics of pollinator perception. For example, orchid pollinia removal was greatest when the mimics were further away from their models (e.g., outside the patch) but within a maximum distance of 10 m from the model patch. Because the pollinia removal of deceptive species can be subject to both competition and facilitation effects depending on the density of rewarding (Julliet et al., 2007) and conspecific plants (Duffy & Stout, 2011) the competition orchids experienced within the patch of floriferous pea plants would have been at its strongest (Figure 3b). However, when we accounted for both floral density of conspecific and model plants along a continuous and wider spatial scale (Figure S2), the pollinia removal pronouncedly declined at distances >15 m from model plants. At such distances, the orchids no longer had to contend with the peas for pollinators' attention, but the beneficial effect of facilitation between the plant species also disappeared. Therefore, the importance of exaggerated UV reflectance in attracting pollinators from a range of several meters can be missed and/or mistakenly dismissed if not measured at the scale at which it has its strongest ecologically relevant effect. Such a long‐range signal might not be suspected considering the typical acuity range of bee‐chromatic vision for stationary stimuli within the confined space of a Y‐maze (Giurfa et al., 1996). Overall, our results support the hypothesis that the functional role of UV reflectance signaling is contingent on the relative distance between deceptive and rewarding species and their pollinators; the distance described here operates at spatial scales of meters, which are much greater than expected for floral colors. The terminal position of the outer petals on a long‐stemmed plant (Figure 1a) likely promotes (wind) movement of this exaggerated UV signal that can be even better perceived from afar by foraging bees (Brock et al., 2016; Stojcev et al., 2011) by acting as a “flag signal.”

Contributing to a range of floral displays aimed at pollinator senses, UV reflectance acts as an important visual cue in many flowering plant species (Johnson & Andersson, 2002; Klomberg et al., 2019). The high UV reflectance of *Diuris* outer petals enables bees to find these relatively scarce flowers from a distance of meters. Selection may favor deceptive floral displays capable of longer‐range UV signaling that help pollinators such as solitary bees to locate flowers in habitats where the distribution of rewarding model flowers is patchy, explaining why relatively large, salient UV signals with high background contrast have evolved in the mimic (Rohde et al., 2013). By revealing that floral salient UV displays are efficiently used by bees not only at the very close ranges already well‐documented but also from further afield at an optimal distance, we may explain how plant deception succeeds despite imperfect floral mimicry. These findings invite us to extend our understanding of the adaptive significance of UV reflectance and salient signaling that plants display in a captivating phenomenon such as floral mimicry and more general in nature.

## AUTHOR CONTRIBUTIONS

**Daniela Scaccabarozzi:** Conceptualization (lead); data curation (lead); funding acquisition (equal); investigation (lead); methodology (equal); resources (equal); supervision (equal); validation (equal); visualization (equal); writing – original draft (lead); writing – review and editing (equal). **Klaus Lunau:** Conceptualization (equal); data curation (equal); methodology (equal); writing – original draft (equal); writing – review and editing (equal). **Lorenzo Guzzetti:** Data curation (equal); formal analysis (equal); writing – review and editing (equal). **Salvatore Cozzolino:** Conceptualization (equal); methodology (equal); supervision (equal); visualization (equal); writing – original draft (equal); writing – review and editing (equal). **Adrian G. Dyer:** Conceptualization (equal); methodology (equal); validation (equal); visualization (equal); writing – review and editing (equal). **Nicola Tommasi:** Methodology (equal); writing – review and editing (equal). **Paolo Biella:** Writing – review and editing (equal). **Andrea Galimberti:** Methodology (equal); validation (equal); visualization (equal); writing – review and editing (equal). **Massimo Labra:** Validation (equal); writing – review and editing (equal). **Ilaria Bruni:** Writing – review and editing (equal). **Giorgio Pattarini:** Methodology (equal); writing – review and editing (equal). **Mark Brundrett:** Methodology (equal); writing – review and editing (equal). **Monica Gagliano:** Conceptualization (equal); methodology (equal); supervision (equal); validation (equal); writing – original draft (equal); writing – review and editing (equal).

## CONFLICT OF INTEREST

The authors declare no competing interests.

## Supporting information


Appendix S1

Data S1

Data S2

Data S3

Data S4

Data S5

Data S6

## Data Availability

Data needed to evaluate the conclusions in the paper are presented in the Supporting Information.
