# Supplementary material for: Mimicking orchids lure bees from afar with exaggerated ultraviolet signals
Source: Ecol Evol. 2023 Jan 29;13(1):e9759. doi: 10.1002/ece3.9759 (PMC9884568; doi:10.1002/ece3.9759)
Supplement: Supplementary file 1 — Appendix S1 [file ECE3-13-e9759-s004.docx]

**Supporting Information**

Title: Mimicking orchids lure bees from afar with exaggerated ultraviolet signals

**This file includes:**

Figures and legends S1, S2

Supplementary text

Table S1

Legends for Data sources S1 to S6

**
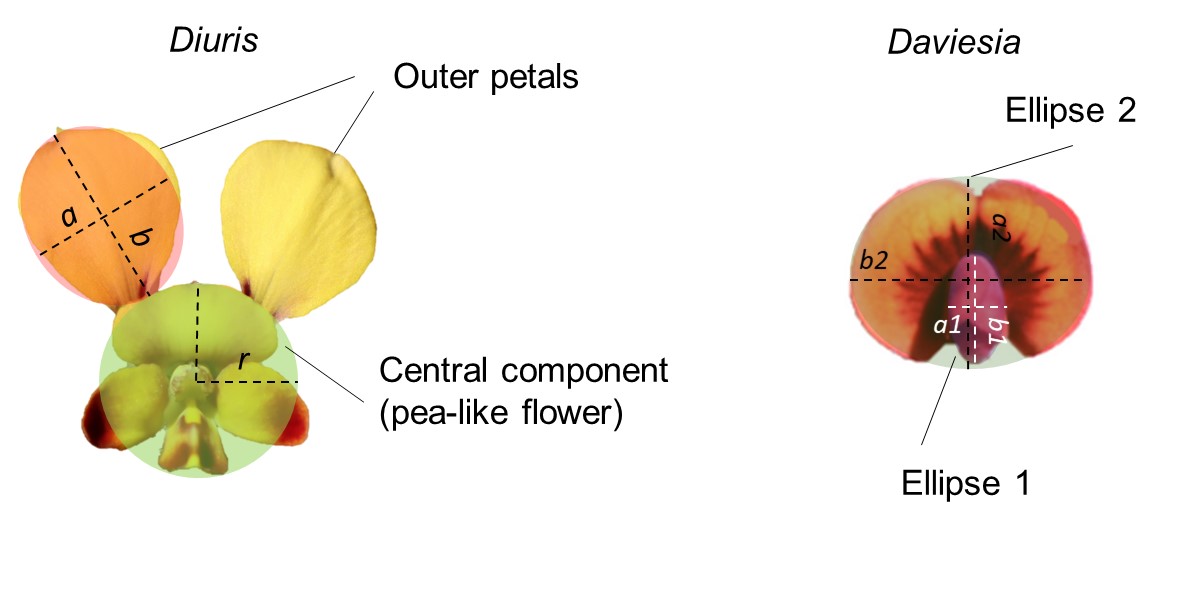
**

**Fig. S1.** Morphological components of *Diuris* and *Daviesia* flowers. The height (a) and width (b) of *Diuris* outer petals forming an approximated ellipse and the radius (r) of its central component (forming an approximated circle) comprised of dorsal sepal plus labellum and labellum lobes. The width (a1) height (b1) of the wing petals comprising the keel edge and the height (a2) width (b2) of *Daviesia* standard petals formed approximated ellipses 1 and 2, respectively.
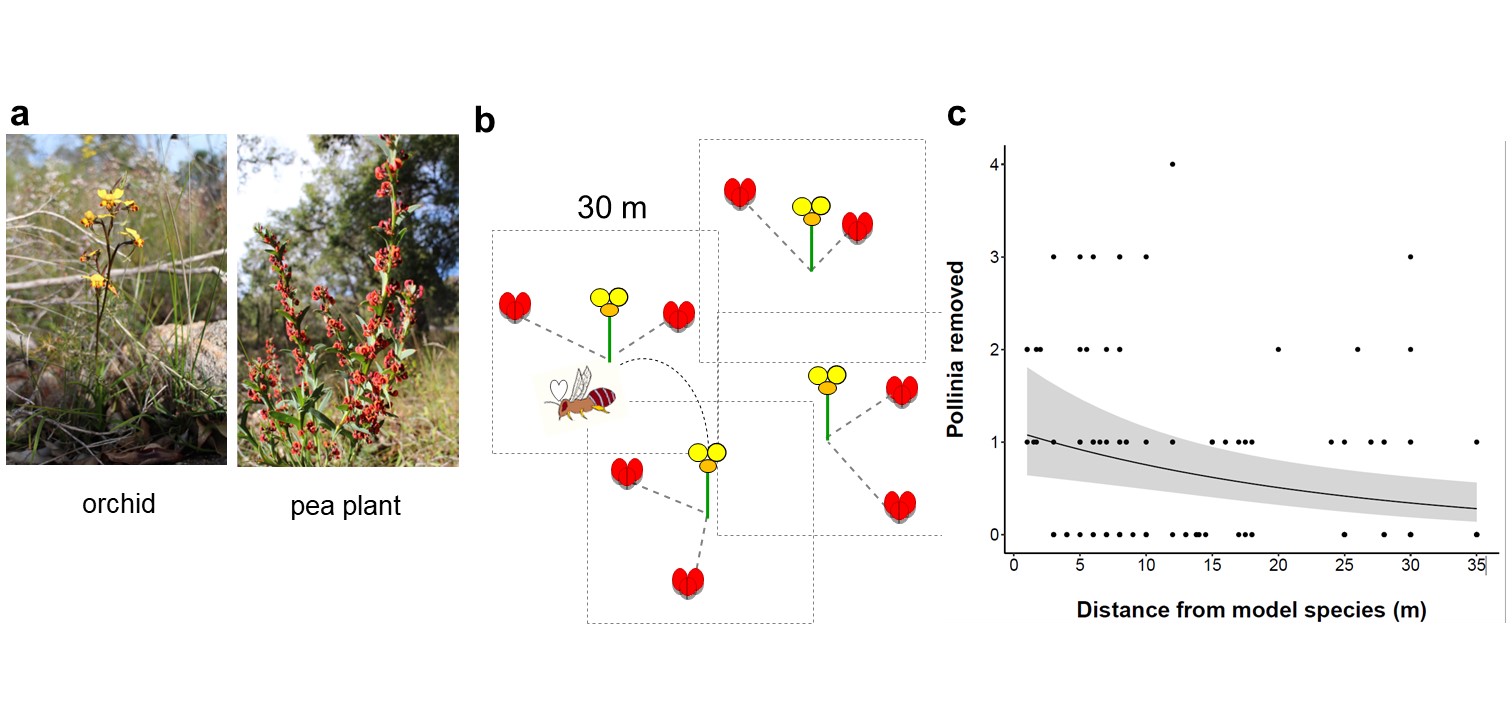


**Fig. S2.** Orchid pollination success relative to distance from model pea plants. (a-b) The effect of relative distance between an individual orchid (yellow flower) and the surrounding pea models (red flower) was quantified within 30 x 30 m quadrat centered on the orchid plant (N = 122 orchids across 5 populations). (c) The number of pollinaria removed from the orchid flowers decreased significantly with the orchids’ distance from the pea model (χ² =10.34, p = 0.001).

**Supplementary Information Text**

**Methods S1**

**Testing the effect of the ultraviolet reflectance screening solution on pollinator visits.** This test was done to isolate the effect of the UV screen spray as attractive or repellent agent to bees, to rule out the possibility that the spray scent would affect the outcome of UV manipulation experiments. The effect of the UV reflectance screening solution (Kinesys, Canada) on the number of *Trichocolletes* bee visits to *Diuris* orchids was tested using choice experiments conducted during the foraging peak of pollinators (11.00 am -1.00 pm) over two days. Two picked inflorescences with identical number of flowers were presented in proximity to model plants according to rotating arrays methodology (Scaccabarozzi et al., 2020a). One of the two inflorescences was treated by applying the screening solution at the base of the corolla, whilst the other one was used as control (non-treated). Number of bees approaching the inflorescences with treated and untreated flowers was recorded for 16 trials and subsequently tested by a Generalized Linear Model with a Bernoulli distribution of the response variable. We found no difference between the number of *Trichocolletes* bees visiting treated (n = 29) and non-treated orchid flowers (n = 31; χ² = 1.11, p = 0.291). We concluded that the screening solution had no effect in attracting to or repelling the pollinators from orchid flowers and thus, suitable for the UV manipulation experiments conducted in the current study.

**Supplementary Information Text**

Methods S2

Conversion of photos of *Diuris* sp. and *Daviesia* sp. in false colour photography

False colour photography in ‘bee view’ format was used to reveal the overall colour pattern perceived by bees in treated (i.e., application of the UV screen solution) and untreated outer petals of *Diuris* flower and untreated *Daviesia* flower. Each flower was photographed in colour (i.e., human view) and UV using a modified Panasonic BMC-G3 camera with a UV-transmitting Ultra-achromatic-Takumar 20 mm F/4 lens made of fused quartz. The white balance was set separately for the colour and UV-photography using a white Teflon disc as a control under same light conditions. The images, taken from the same position and within 10 sec intervening time, were converted into false colour images that comprise the UV, blue and green wavelengths perceived by bees. Image assemblage in bee view was obtained by splitting the colour and the UV-photo into the three camera colour channels, namely blue, green and red. The red channel was discarded from the colour photo and green and red channels were discarded from the UV-photo as in Lunau et al. (2021). In the false colour photos UV wavelengths are represented as blue, blue as green and green as red wavelengths as a standard method for colour image translation to represent bee vision. In bee view, *Diuris* petals treated with the UV screen solution exhibited a different coloration (orange, characterised by green wavelengths) compared to untreated petals (purple, characterized by UV wavelengths and green wavelengths).

**Table S1.** List of the five population sites of *Diuris brumalis* surveyed in this study.

| **Population number** | **Site** | **Latitude, longitude** |
| --- | --- | --- |
| 1 | Lesmurdie - Canning Rd | 32°01'45.6'' °S, 116°06'01.3'' °E |
| 2 | Lesmurdie - Canning Rd | 32°01'41.9'' °S, 116°05'41.7'' °E |
| 3 | Lesmurdie - Canning Rd | 32°01'43.8'' °S, 116°05'12.1'' °E |
| 4 | Lesmurdie - Canning Rd | 32°01'43.2'' °S, 116°04'51.9''° E |
| 5 | Lesmurdie - Canning Rd | 32°01'39.8'' °S, 116°04'45.3'' °E |

**Legends for Data S1 to S6**

**Data S1. Morphological measurements of *Diuris* and *Daviesia* floral components and UV salient signal ratio calculation*.*** Flower measurements (a, b, r) taken on *Diuris* for calculating the area of the geometric figures (ellipse and circle) that approximate the area of the flower components (see Fig. S1). a, b: major and minor axis of the outer petal; r: distance between the pollinaria centre and the top edge of the dorsal sepal. The ellipse approximates the outer petal area that has been duplicated for estimating the total area of the external flower component, formed by the two outer petals. Flower measurements (a1, b1, a2, b2) taken on *Daviesia* for calculating the area of the geometric figures (ellipse 1 and 2) that approximate the area of the flower components (see Fig. S1). a1, b1: major and minor axis of the wing petals comprising the keel edge. a2, b2: major and minor axis of the two standard petals. Calculations of UV salient signal ratio with total surface of flowers (comprising all flower components) and total surface of flowers reflecting more than 10% in UV, according to cut value by Dyer (1996) for Australian plants. *between 300 and 400 nm (full spectra reflectance curve above 10%); ¹outer petals and dorsal sepal ²: standard petal; *Diuris* dorsal sepal: estimated as semi-circle area of the flower central component in Fig. S1. *Daviesia* standard petals: estimated by subtracting the area of Ellipse 1 to the area of Ellipse 2 in Fig. S1.

**Data source S2. Spectral measurements of the floral components of *Diuris brumalis* and *Daviesia decurrens.*** Means and standard deviation of colour reflectance for *Diuris* and *Daviesia*. Means are based on colour measurements across flower components of six individual plants.

**Data source S3. Testing the effect of the UV screening spray on the attraction of *Trichocolletes* bees.** Number of bees approaching *Diuris* inflorescences with UV treated and untreated flowers.

**Data source S4**. **Model-mimic distance experiment.** Testing whether *Diuris* pollination success varies depending on the distance to the model pea plants.

**Data source S5. In and Out experiment.** First field UV manipulation experiment, testing that UV reflectance enhances orchid pollination success when out from peas patch.

**Data source S6.** **Distance from orchids to model plants as continuous variable.** Second field UV manipulation experiment, testing that by displaying an exaggerated version of *Daviesia*’s attractive UV reflectance, *Diuris* benefits from pollinators that mistake it for the rewarding model from afar.
